# Angiomyofibroblastoma as a rare cause of vulvar mass: A case report and literature review

**DOI:** 10.1002/ccr3.7971

**Published:** 2023-09-25

**Authors:** Negar Einafshar, Mahta Shari’at Moghani, Mahsa Radboy, Tooraj Zandbaf

**Affiliations:** ^1^ Innovative Medical Research Center Faculty of Medicine Mashhad Medical Sciences Islamic Azad University Mashhad Iran; ^2^ Department of General Surgery Faculty of Medicine Mashhad Medical Sciences Islamic Azad University Mashhad Iran

**Keywords:** Angiomyofibroblastoma, case Report, vulvar mass, vulvar neoplasm

## Abstract

**Key Clinical Message:**

Angiomyofibroblastoma is a benign soft tissue tumor and a form of genital stromal mesenchymal tumor that primarily affects the vulva. It could possibly affect the reproductive‐aged women's lower genital tract (vagina).

**Abstract:**

Angiomyofibroblastoma is a rare benign soft tissue tumor primarily affecting the vulva in reproductive‐aged women. We report a 67‐year‐old female complaining of a painless mass in her right vulva spreading to the right inguinal region over the past 2 years. The first clinical impression was a canal of Nuck hernia, diagnostic laparoscopy was planned to rule hernia out. The vulvar mass was excised, and a histopathology examination revealed Angiomyofibroblastoma.

## Graphical abstract

A mass in the right major labia spreading to the right inguinal.

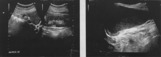

## 1 INTRODUCTION

Angiomyofibroblastoma (AMF) is a benign soft tissue tumor and a form of genital stromal mesenchymal tumor that primarily affects the vulva. It could possibly affect the reproductive‐aged women's lower genital tract (vagina). Clinically, it frequently appeared as a benign, painless enlargement of the vulva, but rarely other manifestations such as foul‐smelling secretions, menorrhagia, and dyspareunia have also been seen. This tumor's first case series was published by Fletcher et al.^1^, ^2^, ^3^, ^4^, ^5^

In this article, we report a case of AMF. Toward a better understanding of this tumor and its histopathology as well as an investigation of its differential diagnosis, we manage to conduct this article. Our work has been documented according to the CARE guidelines.

## 2 CASE PRESENTATION

### 2.1 Presentation

**FIGURE 1:**
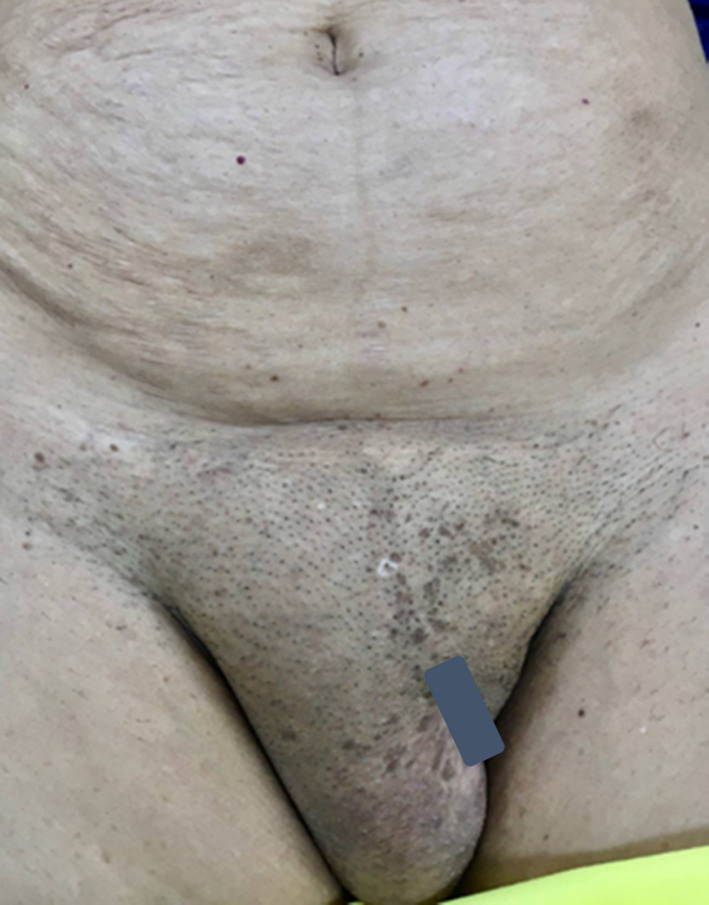
The painless mass in the right major labia with extension to the right groin.

A 67‐year‐old white married female was referred by a gynecologist to our department for complaining of a painless mass in her right vulva spreading to the right inguinal region without any other symptoms from the past 2 years (Figure 1). The mass gradually increased in size over 4 months. She had no history of changes in appetite and bowel movements, urinary symptoms, and weight loss. She reported no history of genital infection, gynecologic cancer, and sexually transmitted disease. The patient had four pregnancies and had a history of hysterectomy due to abnormal uterine bleeding in 2016. In addition, the patient reported that she has taken valsartan/amlodipine tablet (80 mg/5 mg) since she is suffering from hypertension. She had no history of using oral contraceptives in the past. She did not smoke or drink alcohol.

**FIGURE 2:**
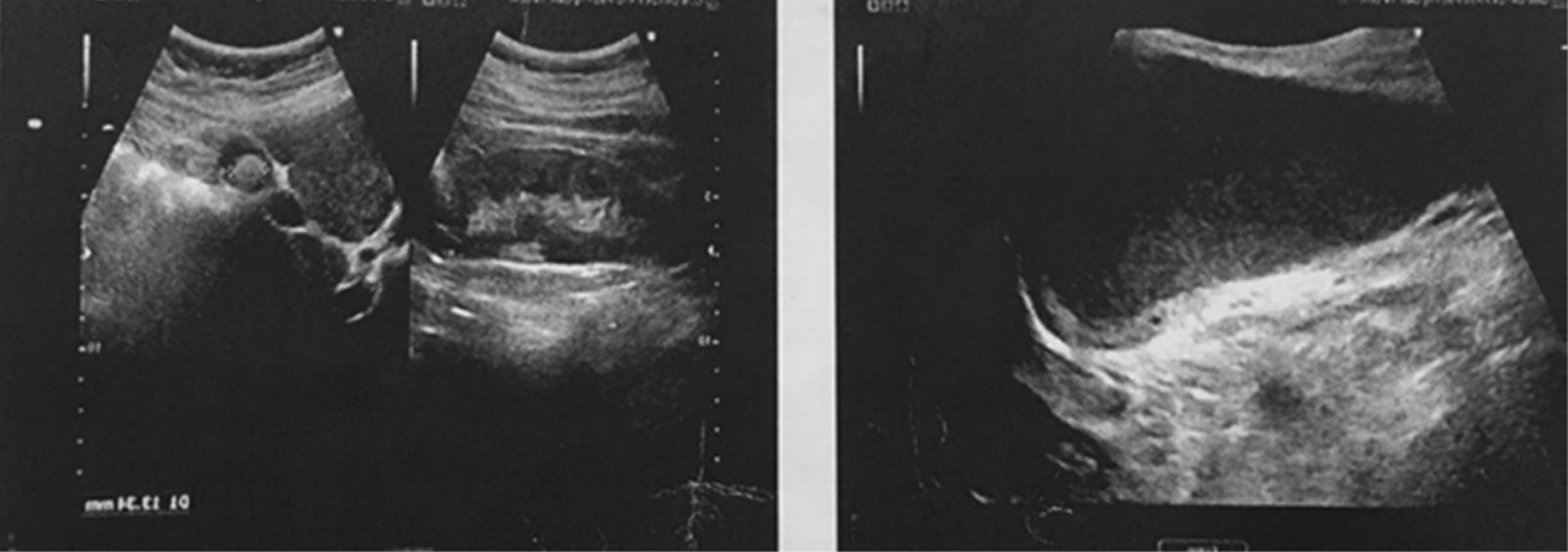
A mass measuring 90*44*55 mm in the right major labia spreading to the right inguinal on ultrasound.

She was systemically well on the examination. Her vital signs and abdominal examination were normal. There was an approximately 12 cm painless mass in the right vulva spread to the right groin. The right groin was quite prominent and the hernia was first considered as a diagnosis. No signs of infection or inflammation and lymphadenopathy were observed. Internal genital organ examination was normal. An ultrasound of soft tissue demonstrated a hypoechoic solid mass with specific borders in size of 90*44*55 mm located in the right major labia which was extended to the right inguinal (Figure 2). In this regard, the report suggested a possible tumor originated from this local soft tissue. Color Doppler assessment showed mild scattered vessels in the peripheral. Laboratory tests were completely normal. The first gynecologist's medical impression was a canal of Nuck hernia. Because the vulval bulge was extended to the groin area, an incarcerated hernia was strongly suspected. Moreover, the features described in the major labia ultrasound indicated the omentum tissue for us.

### 2.2 Surgical procedure

**FIGURE 3:**
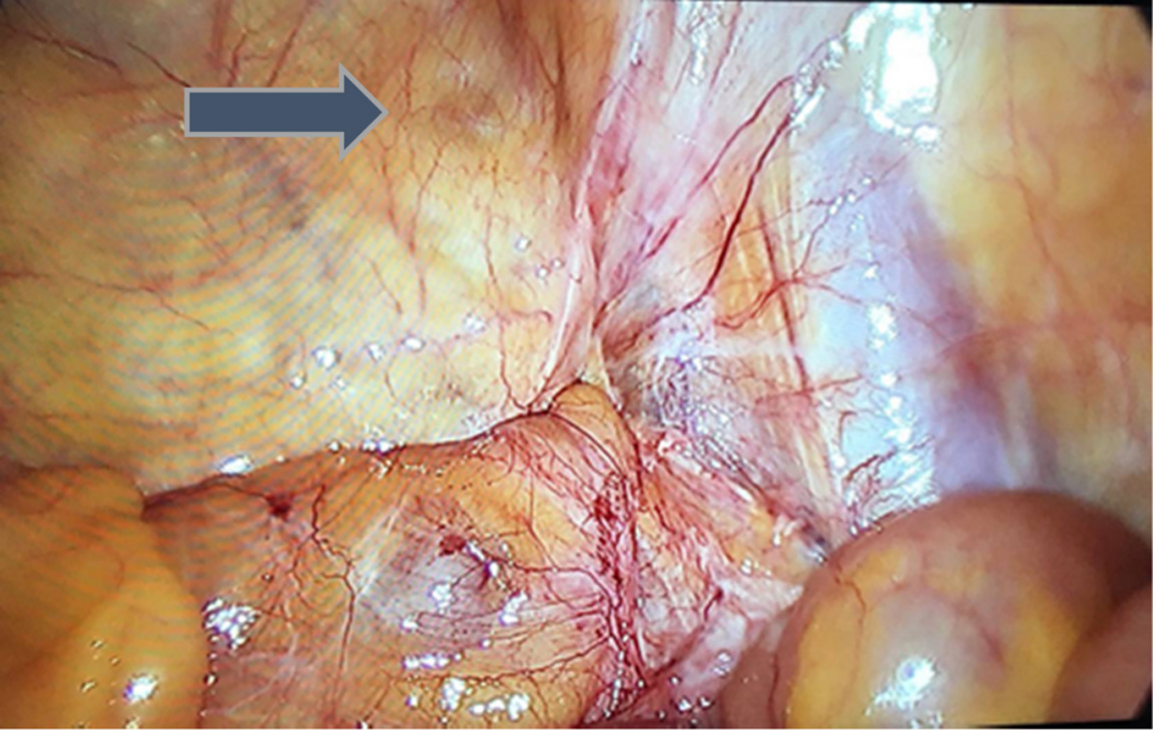
The weakened area in the right inguinal region in laparoscopic view.

**FIGURE 4:**
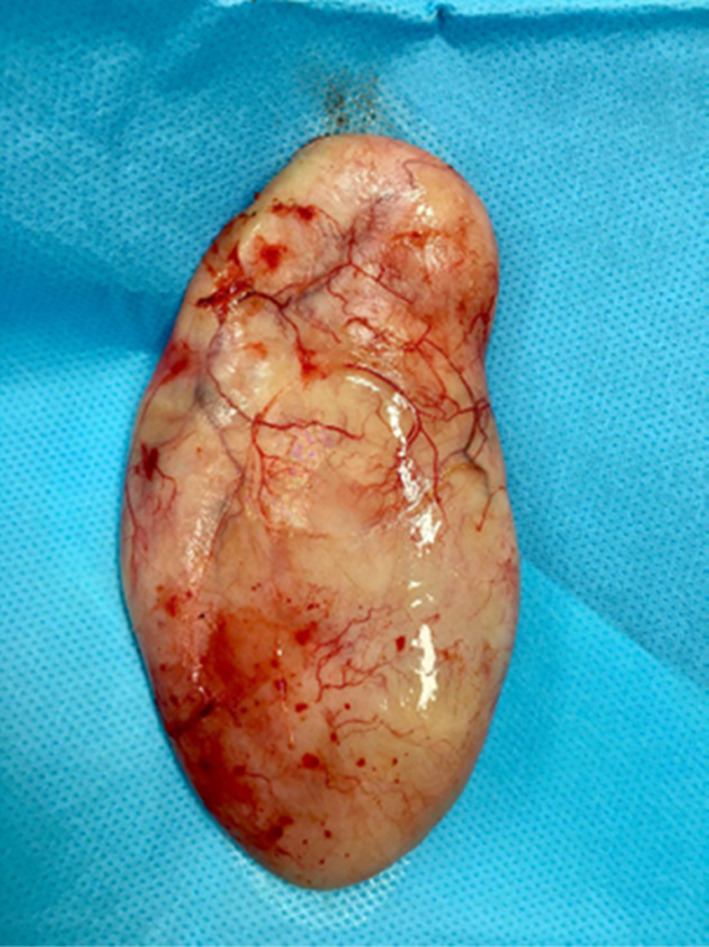
Macroscopic view of the right major labia mass.

The patient was a candidate for a diagnostic laparoscopy. On operation, after anesthesia and prep and drape, a pneumoperitoneum pressure of 12 mm Hg was set up by a Veress needle. A trocar with a diameter of 10 mm was inserted at the superior margin of the umbilicus for the camera. A tow trocar with a diameter of 5 mm was inserted at the external margin of the umbilicus paralleled rectus abdominis on the right and left sides. On abdominal exploration, a weakened area was seen in the right inguinal region (Figure 3). Still, no other pathology was seen in the abdomen and pelvis except for evidence of a previous hysterectomy. An arc incision in the peritoneum in the right inguinal region was made. The preperitoneal space was sharply isolated, and a classical dissection of the inguinal region was performed. However, no hernia was seen in the inguinal, femoral, obturator, and canal of Nuck areas yet. The peritoneum was closed. Then, we decided to undergo surgery on the right major labia. Therefore, an incision was made over the lesion and a 12 cm lump with a well‐defined margin without much adhesion to the surrounding tissue was removed (Figure 4). After performing hemostasis, the subcutaneous and skin were repaired.

### 2.3 Histopathology

The gross examination of the surgical specimen revealed the encapsulated mass with grayish‐white color in appearance. The mass measured 11.5*6.5*3.5 cm and weighed 131 g. The cut section showed a solid grayish‐white homogenous edematous surface with no necrosis. Microscopic examination demonstrated well‐demarcated neoplastic lesions composed of some spindle cells with spindly non‐atypical nuclei, some collagen bundles within the loose edematous stroma, and many thin‐wall small vessels. No necrosis and rare mitosis were found. In immunohistochemical tests, the cells were positive for desmin, smooth muscle actin (SMA), and estrogen receptors (ER). CD34 for spindle cells was negative whereas on thin‐wall vessels was positive. Based on these features, the pathological impression indicated Angiomyofibroblastoma.

### 2.4 Postoperative follow‐up

The patient visited 1 week after surgery. Her vital signs were normal and there was no evidence of hematoma or infection at the surgical site. In an oncology consultation, it was confirmed that the mass is completely benign and there is no need to perform any other treatments. After 3 months, our patient remained well and asymptomatic.

## 3 DISCUSSION AND CONCLUSION

In this report, we discussed AMF of the vulva in a 76‐year‐old woman that was doubted to be a canal of Nuck hernia. The labia majora is the site of the canal of Nuck hernias, which is caused due to the continuous protrusion of the parietal peritoneum into the inguinal canal.^6^ As seen in our case, the canal of Nuck hernias can coexist in the same region as AMF. Therefore, it is sometimes difficult for specialists to diagnose accurately.

Vulvar AMF is an uncommon, painless, and benign mesenchymal neoplasm with a great prognosis. However, it might turn into malignancy.^7^, ^8^, ^9^ The most typical mesenchymal tumor of the lower female genital tract is AMF.^10^ In 1992, Fletcher et al. first documented 10 cases of female vulva AMF.^4^ On average, AMF affects women in their third to fifth decades of life. The patients' ages vary from 17 to 86 (Mean, 45 years),^7^ and diagnosis of AMF should be investigated in women of childbearing age who come with vulvovaginal mass due to a tendency toward the area.^5^, ^11^ Nevertheless, our patient at 67 years is older than this typical age range.

Although speculative, most scholars believe that the disease may originate from stem cells.^12^ Microscopic analysis revealed a well‐demarcated lesion with the presence of numerous thin‐walled blood vessels surrounded by spindle cells. The stroma was edematous, loose, and collagenous. There was no sign of mitosis, necrosis, or neoplasia in the sections analyzed.^5^ According to a study by Nagai et al., AMF's size can vary from 0.5 to 23 cm.^13^ Most of these lesions are <5 cm.^14^, ^15^ AMF grows gradually in size. Therefore, most of the patients already become aware of tumors 1 year in advance.^11^, ^16^ Even Anggraeni et al. reported an AMF case having developed over 10 years.^17^ Because pedunculated AMF is uncommon, only a few cases have been described yet.^7^, ^13^, ^18^

The immunohistochemistry profile of AMFs is nearly positive for vimentin, desmin, progesterone, and estrogen receptor. But SMA expression in the tumor cells varies.^4^, ^7^, ^19^ Although CD34 was not expressed by the tumor cells, the vessels may have expressed some reactivity.^7^, ^20^, ^21^ In our case, the absence of invasive characteristics is consistent with AMF features. Based on AMF Immunohistochemistry characteristics, diagnoses can be expected in our case.

Even though aggressive angiomyxoma (AA) is the first differential diagnosis that comes to mind, other mesenchymal tumors, hydrocele of the canal of Nuck, Bartholin's cyst, leiomyoma, inguinal hernia, cellular angiofibroma, and fibroepithelial stromal polyp need to be considered either.^5^, ^22^, ^23^ Because AA demands more extensive treatment, it must be carefully distinguished from AMF.^14^ AA was introduced first by Steeper and Rosai^23^ characterized by less cellular, larger vessels that may have thick walls and hyaline change, an average size of >5 cm as well as having a short duration of symptoms compared to AMF. Although the majority of AAs induce estrogen and progesterone receptors expression, desmin receptors are sometimes expressed.^24^

Like our case, several investigations claimed that ultrasonography might be useful for the diagnosis of AMF since it can identify the exact borders of AMF. Soft tissue with a hypoechoic mass and a partially visible blood flow signal inside the mass can also be provided by ultrasonography. The definitive diagnosis is finally confirmed by postoperative pathological examination. Currently, no drugs are available to treat AMF, and surgery is preferred.^16^, ^25^ It might be worth noting that AMF has a slight chance of recurrence or spreading.^5^, ^11^, ^26^

Regarding the unknown and different characteristics of vulvar AMF, opinions, and clinical approaches are controversial. For instance, Akhtar et al presented a 25‐year‐old woman with a 1.5–2 cm, painless, cystic lesion over the labia minor that was initially thought to be a Bartholin's cyst which has gradually grown in size in the past 2 months.^27^ Alves Ruas et al. described a 35‐year‐old woman who had painful vulvar swelling with a 7 cm mass in the right labia major for 4 months.^28^ Medical history in the cases mentioned above was not significant. In the end, both had full excision with well‐defined margins. In our case, we investigated a case of 11.5 cm AMF of the vulva in a 76‐year‐old post‐menopausal female with a preoperative diagnosis of the canal of Nuck hernia. The lump was painless, unlike Alves Ruas's study. In contrast to the findings of both studies, our patient had a history of hysterectomy and oophorectomy. According to our initial diagnosis, laparoscopic surgery was eventually performed. However, contrary to expectations, no signs of hernia were seen, and later histopathological analysis confirmed the diagnosis of AMF. To better understand and compare the findings of our study and other similar studies, we compared various parameters and classified them in Table 1.

**TABLE 1:** Clinical findings of nine patients with AMF.

N	Author	Age	Site	Size (cm)	Peduncle	Symptom	Vessel & Stromal Cell Pathology	IHC	Ultrasound
1	Reyna^29^	37	Right	17 × 9	Pedunculated	Painless mass	Hypercellular and hypocellular stromal edema Spindle cells that surround thin‐walled blood vessels	Vimentin+Desmin+ER+PR+	A homogeneous lesion, composed of soft tissue with normal vascularization
2	Pradhan^30^	42	Right	4 × 3	Non‐pedunculated	Swelling	Hypercellular and hypocellular stromal edema capillary sized blood vessels surrounded by spindled stromal cells	Vimentin+CD34^+^ in vessel	N/A
3	Chen^25^	50	Left	2.5 × 2	Non‐pedunculated	Painless mass	Hypercellular and hypocellular regions of oval or epithelioid stromal cells, aggregated around blood vessels	SMA+ER+PR+Vimentin+Desmin−CD34^+^	Elliptic mass with complete border, non‐uniform hypoechoic background, irregularly distributed short‐strip high echo, without any echo attenuation or enhancement on the posterior region
4	Chen^25^	48	Left	3 × 2.2	Non‐pedunculated	Spontaneous pain	Sparse collagen fibers and had small or medium‐sized blood vessels around which the tumor cells were aggregated, spindle‐shaped cells	SMA+ER+PR+vimentin+Desmin+CD34^+^	Soft tissue tumor with healthy blood vessels and homogeneous echo
5	Shetty^5^	40	Left	6 × 2	Non‐pedunculated	Menorrhagia, slow‐growing, painless mass, dyspareunia	Hypercellular and hypocellular areas, comprising spindle cells be clustered around numerous capillary‐sized thin‐walled blood vessels	N/A	Well‐defined heterogeneously iso hypoechoic lesion with multiple dense echoes
6	Anggraeni^17^	22	Left	19 × 14	Non‐pedunculated	Painless mass	Thin‐walled blood vessels surrounded by structurally arranged stromal cells. The tumor contained fatty tissue	SMA+ER+PR+Desmin+CD34^+^	Internal genitalia were within normal limits
7	Nagai^13^	48	Right	23 × 20	Pedunculated	Painless mass	Well‐ circumscribed lesion composed of hyper and hypocellular areas. spindle‐shaped stromal surrounded thin‐walled capillaries	SMA‐ER+PR+Vimentin+Desmin+CD34^−^	N/A
8	Nam^31^	29	Right & left	3 × 10 (right) 5 × 12 (left)	Non‐pedunculated	Painless mass, inability to walk freely due to the massive swelling of both labia	Spindle‐shaped–to‐plump cells in the loose stroma with prominent vascularity were noted	N/A	A solid hypo‐ echogenic mass with peripheral vascularization
9	Park^32^	51	Right	2.5 × 1.5	Non‐pedunculated	Painless mass	Hypercellular and hypocellular edematous areas and the plump stromal cells clustered around abundant blood vessels	ER+PR+Vimentin+Desmin+	A slightly hypoechogenic lesion of 21 mm with posterior shadowing. Assumed to be a vulvar cystic mass
10	Present Case	67	Right	11.5 × 6.5	Non‐pedunculated	Painless mass	Well‐demarcated neoplastic lesions composed of some spindle cells with spindly non‐atypical nuclei, some collagen bundles within the loose edematous stroma, and many thin‐wall small vessels	SMA+ER+Desmin+CD34^+^ in vessel	A hypoechoic solid mass with specific borders, which was in the right major labia spreading to the right inguinal

Due to the small number of patients and limited accessible follow‐up data, the long‐term clinical activity of AMF, the role of hormones on tumor formation, and the consequences of gynecological procedures as risk factors are not yet obvious and further studies need to be done in this field.

## AUTHOR CONTRIBUTIONS

**Negar Einafshar:** Writing – original draft. **Mahta Shari'at Moghani:** Writing – original draft. **Mahsa Radboy:** Writing – original draft. **tooraj Zandbaf:** Conceptualization; supervision; writing – review and editing.

## CONFLICT OF INTEREST STATEMENT

The authors declare that they have no competing interests.

## CONSENT

The patient's written consent was obtained for the publication of this case report.

## Data Availability

The data that support the findings of this study are available from the corresponding author upon reasonable request.
